# Tumour GDF-15 Expression and Clinical Outcomes in Intermediate-Risk Metastatic Clear-Cell Renal Cell Carcinoma Treated with Second-Line Nivolumab

**DOI:** 10.3390/curroncol33090531

**Published:** 2026-09-02

**Authors:** Orhun Akdogan, Betul Ogut, Osman Sutcuoglu, Melike Urganci, Burcu Ulas Kahya, Ipek Isik Gonul, Hatice Azra Begum Salimoglu, Tuba Ugur Tuzcu, Ozan Yazici, Ahmet Ozet, Nuriye Ozdemir

**Affiliations:** 1 Department of Medical Oncology, Faculty of Medicine, Gazi University, 06560 Ankara, Turkeytubaugurtuzcu@gazi.edu.tr (T.U.T.);; 2 Department of Pathology, Faculty of Medicine, Gazi University, 06560 Ankara, Turkey; 3 Department of Internal Medicine, Faculty of Medicine, Gazi University, 06560 Ankara, Turkey

**Keywords:** GDF-15, renal cell carcinoma, nivolumab, immune checkpoint inhibitor, immunohistochemistry, tissue biomarker, cachexia

## Abstract

**Background:** Immune checkpoint inhibitors have improved outcomes in metastatic clear-cell renal cell carcinoma (mRCC), yet clinically applicable tissue biomarkers remain limited. Growth differentiation factor-15 (GDF-15) promotes tumour immune evasion and has emerged as a potential therapeutic target in immuno-oncology. We evaluated the prognostic significance of tumour GDF-15 expression in patients with intermediate-risk clear-cell mRCC treated with second-line nivolumab. **Methods:** Forty-six patients with intermediate-risk clear-cell mRCC who received nivolumab after one line of tyrosine kinase inhibitor therapy were retrospectively evaluated. Tumour GDF-15 expression was assessed by immunohistochemistry and classified as low (0–1+) or high (2–3+). Objective response rate (ORR), progression-free survival (PFS), overall survival (OS), and the development of cancer-associated cachexia were compared between expression groups. **Results:** High tumour GDF-15 expression was observed in 23 patients (50%). ORR was significantly higher in the low-expression group than in the high-expression group (57% vs. 26%, *p* = 0.036). Low tumour GDF-15 expression was associated with significantly longer PFS (24.5 vs. 7.5 months; HR 0.38, 95% CI 0.18–0.80; *p* = 0.009) and OS (28.6 vs. 12.6 months; HR 0.43, 95% CI 0.19–0.98; *p* = 0.041). The association with OS remained significant after adjustment for age. The frequency of cancer-associated cachexia did not differ according to tumour GDF-15 expression (43% vs. 35%, *p* = 0.546). **Conclusions:** Low tumour GDF-15 expression was associated with better objective response and longer progression-free and overall survival in patients with intermediate-risk metastatic clear-cell renal cell carcinoma treated with second-line nivolumab, with the association with overall survival remaining significant after adjustment for age. Tumour GDF-15 represents a promising tissue biomarker for prognostic risk stratification and warrants validation in larger prospective studies.

## Simple Summary

Immune checkpoint inhibitors are commonly used to treat metastatic kidney cancer, but patients receiving the same treatment may experience markedly different outcomes. Routine tissue markers that provide additional prognostic information are lacking. Growth differentiation factor-15 (GDF-15) is involved in tumour immune regulation and cancer-associated weight loss. We examined tumour samples from 46 patients with metastatic clear-cell renal cell carcinoma who received second-line nivolumab. Patients with low tumour GDF-15 expression responded more frequently and had longer progression-free and overall survival than patients with high expression. These findings support further investigation of tumour GDF-15 as a candidate prognostic biomarker alongside established clinical risk factors. Larger prospective studies are required before this marker can be incorporated into clinical practice.

## 1. Introduction

Immune checkpoint inhibitors have changed the treatment of metastatic clear-cell renal cell carcinoma (mRCC). Combination regimens are now preferred in the first line for most patients, but single-agent nivolumab remains in routine use in several settings, particularly where access to combination immunotherapy is restricted and after progression on a tyrosine kinase inhibitor (TKI) [1]. Treatment decisions still rest mainly on clinical prognostic models such as the International Metastatic Renal Cell Carcinoma Database Consortium (IMDC) classification and its predecessors [2,3]. These models summarise patient and disease characteristics rather than tumour biology, and they were developed before checkpoint inhibition entered practice. Access adds a further constraint, since financial and structural barriers limit the use of immunotherapy in many health systems [4]. An inexpensive tissue biomarker that provides prognostic information could therefore be clinically useful.

Growth differentiation factor-15 (GDF-15) is a divergent member of the transforming growth factor-beta superfamily with roles in inflammation, metabolism and tumour progression [5]. Experimental evidence suggests that GDF-15 contributes to immune escape by impairing dendritic cell maturation, limiting antitumour T-cell activity and promoting an immunosuppressive tumour microenvironment [6,7]. GDF-15 also acts through the brainstem receptor GFRAL to suppress appetite and contribute to cancer-associated weight loss [8,9,10]. The clinical relevance of this pathway has increased following the development of GDF-15-directed therapies. In the phase 1–2a GDFATHER study, visugromab combined with nivolumab showed antitumour activity in patients with solid tumours that had progressed during prior anti-PD-1 or anti-PD-L1 treatment [11]. In addition, ponsegromab improved body weight and appetite-related outcomes in patients with cancer cachexia [12].

Most clinical data on GDF-15 in renal cell carcinoma come from serum measurements. Traeger et al. found that high serum concentrations tracked with metastasis, relapse and shorter survival in a cohort of 94 patients with RCC [13], and comparable associations have been described in other tumour types [14]. Serum levels are not tumour-specific, however. They rise with ageing and impaired renal function [15], chronic lung disease [16], heart failure [17], and systemic inflammation of any cause [5], all of which are common in this population. Expression measured directly in tumour tissue avoids much of that noise and should reflect the local microenvironment more closely. Tissue GDF-15 has been examined in non-small cell lung cancer, where high expression was linked to poorer outcomes after immunotherapy [18] and to cachexia [19], but it has not been studied in patients with RCC receiving checkpoint inhibitors.

Cachexia is relevant here for a second reason: patients who are cachectic respond less favourably to checkpoint inhibitors and die sooner [20,21]. Whether tumour GDF-15 expression and clinical cachexia reflect the same process is unresolved. We therefore evaluated the association between tumour GDF-15 expression and clinical outcomes in a homogeneous cohort of patients with intermediate-risk clear-cell mRCC treated with second-line nivolumab after one prior tyrosine kinase inhibitor. The primary objectives were to examine progression-free and overall survival according to tumour GDF-15 expression. Objective response and the relationship between tumour GDF-15 expression and cancer-associated cachexia were evaluated as secondary outcomes.

## 2. Materials and Methods

### 2.1. Study Design and Patients

This single-centre retrospective cohort study was conducted at the Oncology Clinic of Gazi University and is reported in accordance with the STROBE statement for observational studies.

**Figure 1:**
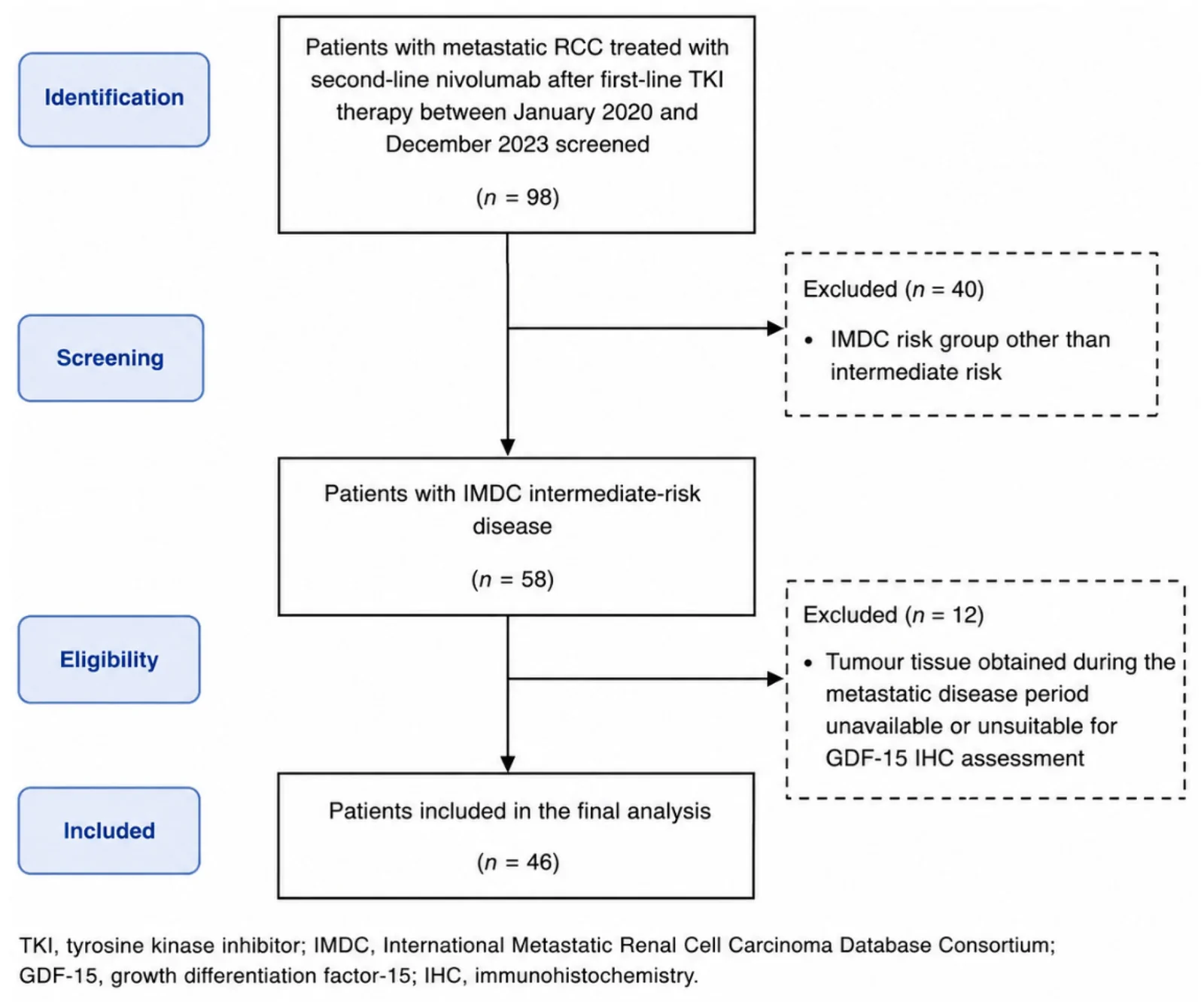
STROBE flow diagram of patient selection.

Adults aged 18 years or older with metastatic clear-cell RCC treated between January 2020 and December 2023 who received second-line nivolumab following first-line TKI therapy were screened. To obtain a clinically homogeneous cohort, eligibility was restricted to patients with intermediate-risk disease according to the IMDC classification and an Eastern Cooperative Oncology Group performance status (ECOG PS) of 0 or 1. Among 98 patients screened, 58 had intermediate-risk disease. Of these, 12 were excluded because tumour tissue obtained during the metastatic disease period and suitable for GDF-15 immunohistochemical assessment was unavailable, leaving 46 patients for the final analysis (Figure 1). Nivolumab was administered intravenously at a dose of 240 mg every 2 weeks until disease progression, unacceptable toxicity, or treatment discontinuation for another clinical reason.

Availability of tumour tissue obtained during the metastatic disease period and suitable for GDF-15 immunohistochemical assessment was required for inclusion. No patients were excluded because of early progression, early death, or insufficient clinical follow-up.

Clinical variables including age, sex, stage, metastatic sites, comorbidities, treatment history, and survival outcomes were retrieved from electronic medical records. Cancer-associated cachexia was evaluated as a secondary outcome. Cachexia was assessed retrospectively using serial body weight and body mass index (BMI) measurements recorded before each nivolumab treatment cycle during routine clinical follow-up. Patients were classified as having cachexia according to the international consensus definition as either unintentional body weight loss exceeding 5% during treatment or body weight loss exceeding 2% in patients with a BMI below 20 kg/m^2^ [22,23]. Because standardised radiological assessment of skeletal muscle mass was unavailable, sarcopenia-based diagnostic criteria were not applied.

### 2.2. Immunohistochemistry

Formalin-fixed, paraffin-embedded tumour specimens obtained during the metastatic disease period and before initiation of first-line TKI therapy were retrieved from the pathology archives. Metastatic tumour specimens from lymph node or lung sites were preferentially used when available. In the six patients with bone-only metastatic disease, bone specimens were not used for GDF-15 assessment; instead, the primary renal tumour specimen obtained during the metastatic disease period was analysed. When more than one eligible pretreatment tumour specimen was available, the most recently obtained specimen was selected for immunohistochemical evaluation. Two experienced pathologists (I.I.G. and B.O.), blinded to clinical outcomes, reviewed all specimens to confirm the presence of viable tumour and selected representative blocks with high tumour-cell content and minimal necrosis.

After deparaffinisation and heat-induced epitope retrieval, tissue sections were incubated with a mouse monoclonal anti-GDF-15 antibody (clone G-5, sc-377195; Santa Cruz Biotechnology, Dallas, TX, USA) at a 1:100 dilution. Staining was performed on a BenchMark ULTRA automated staining platform (Ventana Medical Systems, Inc., Tucson, AZ, USA) using CC1 antigen retrieval and the ultraView DAB detection system, with primary antibody incubation for 32 min. Placental tissue was used as an external positive control, and a negative control was included in each staining run to assess non-specific staining. Positive GDF-15 expression was defined as specific GDF-15 staining in tumour cells.

**Figure 2:**
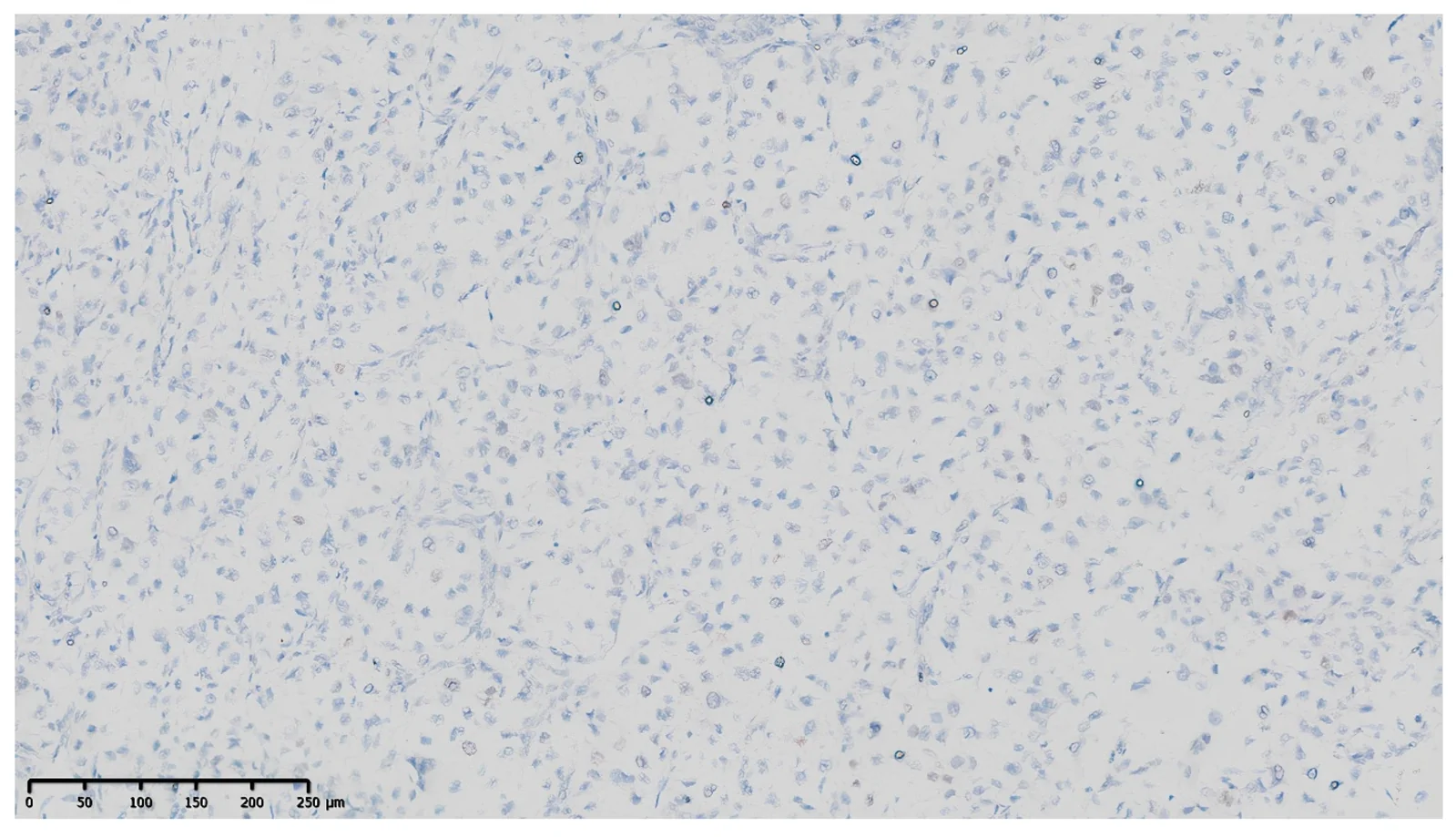
Low tumour GDF-15 expression. Representative immunohistochemistry showing absent-to-faint GDF-15 staining (score 0 to 1+). Scale bar, 250 micrometres.

**Figure 3:**
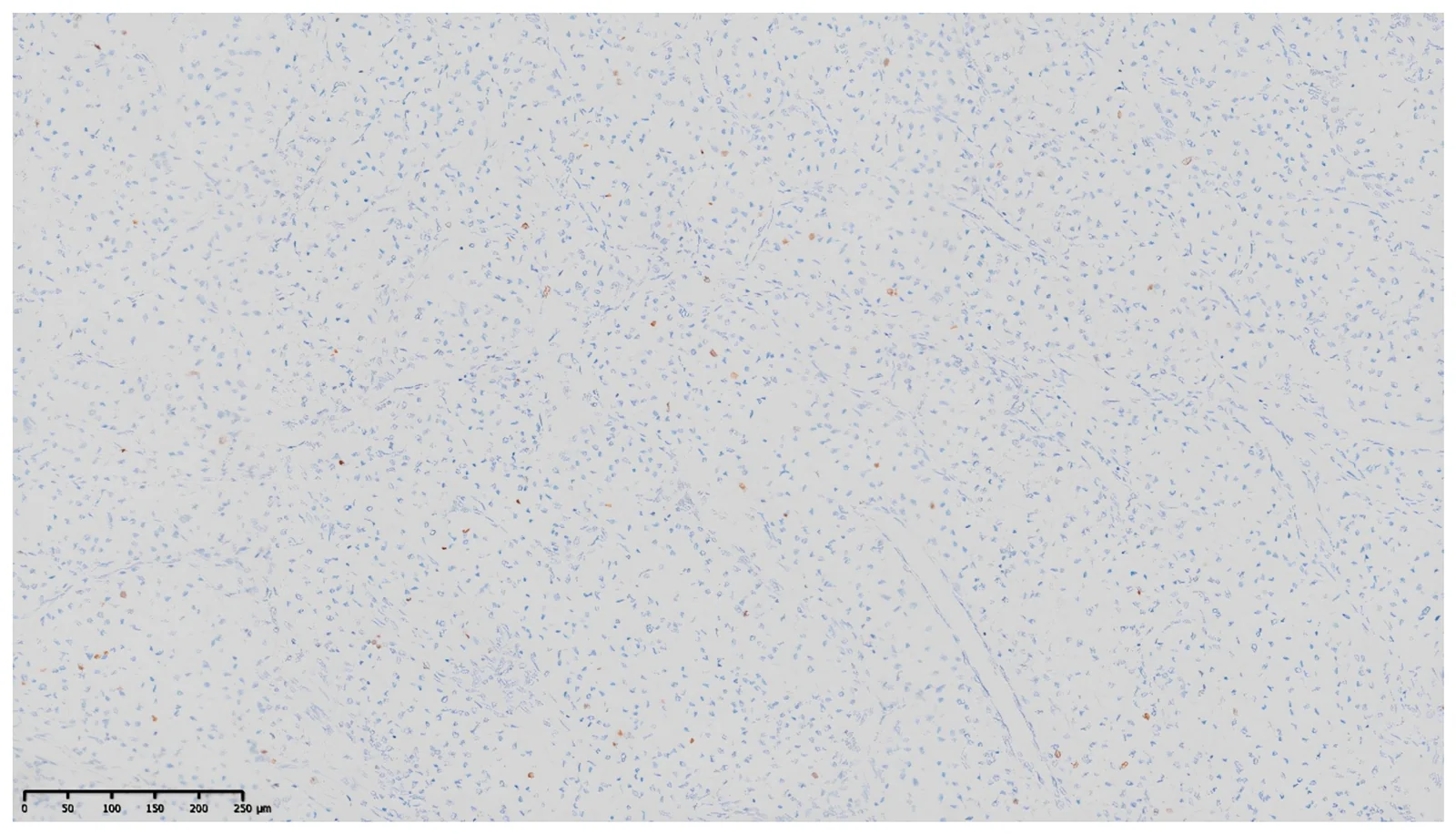
High tumour GDF-15 expression. Representative immunohistochemistry showing diffuse GDF-15 staining (score 2 to 3+). Scale bar, 250 micrometres.

Staining was scored independently by the two pathologists using a semi-quantitative system adapted from Morita-Tanaka et al. [19]: 0, no staining; 1+, partial staining; 2+, diffuse weak-to-moderate staining; and 3+, diffuse strong staining. Interobserver agreement for the four-level ordinal GDF-15 score was excellent (linear weighted Cohen’s κ = 0.98). The single case with discordant initial scoring (2+ versus 3+) was jointly re-evaluated by both pathologists, and a consensus final score of 2+ was assigned. This discrepancy did not affect classification into the low- or high-expression group. Tumours were classified as having low (0–1+; Figure 2) or high (2–3+; Figure 3) GDF-15 expression according to a pre-specified cut-off consistent with previous work [18].

### 2.3. Outcomes

The primary endpoints were progression-free survival (PFS) and overall survival (OS). PFS was measured from nivolumab initiation to radiological progression or death from any cause, whichever occurred first. OS was measured from nivolumab initiation to death from any cause. Patients without an event were censored at the date of last follow-up. Secondary endpoints were objective response rate (ORR) and the development of cancer-associated cachexia during nivolumab treatment. Treatment response was obtained retrospectively from routine clinical and radiological records, with best overall response categorised according to RECIST version 1.1 [24]. ORR was defined as the proportion of patients achieving complete or partial response. No dedicated central or blinded retrospective radiological re-review was performed for this study.

### 2.4. Statistical Analysis

Categorical variables are summarised as counts and percentages, and continuous variables as mean with standard deviation or median with range. Groups were compared with the chi-square or Fisher exact test for categorical variables and with Student’s *t* test or Wilcoxon rank-sum test for continuous variables, as appropriate. Survival was estimated by the Kaplan–Meier method and compared with the log-rank test. Hazard ratios (HRs) with 95% confidence intervals (CIs) were derived from Cox proportional-hazards models, and the proportional-hazards assumption was checked graphically. Variables with *p* < 0.10 on univariable analysis entered the multivariable model. A two-sided *p* value below 0.05 was considered significant. No adjustment was made for multiple comparisons; therefore, secondary and exploratory analyses should be interpreted accordingly. Analyses were performed with SPSS version 27 (IBM, Armonk, NY, USA). Because cachexia was assessed during nivolumab treatment rather than at treatment initiation, it was considered a time-emergent secondary outcome and was not entered as a fixed baseline covariate in the multivariable Cox models. The retrospective assessment schedule did not permit reliable determination of the exact onset of cachexia; therefore, survival analyses according to cachexia status were not performed.

## 3. Results

### 3.1. Patient Characteristics

Forty-six patients with intermediate-risk clear-cell mRCC treated with second-line nivolumab were included. Median age was 62.7 years (range, 42 to 78 years) and 37 patients (80%) were men. At the start of nivolumab, 9 patients (20%) had an ECOG PS of 0 and 37 (80%) had an ECOG PS of 1. Thirty patients (65%) had received sunitinib and 16 (35%) pazopanib as first-line TKI. Six patients (13%) had bone-only metastases, 27 (59%) visceral-only metastases and 13 (28%) metastases at multiple sites. Thirty-one patients (67%) had one adverse IMDC risk factor and 15 (33%) had two adverse IMDC risk factors. Among patients with low GDF-15 expression, 15 (65%) had one and 8 (35%) had two adverse risk factors, compared with 16 (70%) and 7 (30%), respectively, in the high-expression group.

The final distribution of GDF-15 staining scores was 0 in 18 patients (39.1%), 1+ in 5 (10.9%), 2+ in 14 (30.4%), and 3+ in 9 (19.6%), resulting in 23 patients each in the low- and high-expression groups. Age, sex, ECOG PS, prior TKI, chronic kidney disease, metastatic disease distribution, and the development of cachexia did not differ significantly between the two GDF-15 expression groups (Table 1).

### 3.2. Objective Response

The distribution of best overall response differed between the groups (*p* = 0.033). A complete or partial response was recorded in 13 of 23 patients with low GDF-15 expression (ORR, 56.5%; 95% CI, 34.5–76.8%) and in 6 of 23 patients with high expression (ORR, 26.1%; 95% CI, 10.2–48.4%). The odds of achieving an objective response were higher in the low-expression group (OR, 3.68; 95% CI, 1.06–12.77; *p* = 0.036). Progressive disease as best response was twice as frequent in the high-expression group (12 patients, 52%) as in the low-expression group (6 patients, 26%) (Table 1).

### 3.3. Progression-Free Survival

Median follow-up was 26.4 months (range, 5.8 to 52.9 months). At data cut-off, 26 PFS events had occurred: 12 of 23 patients (52.2%) in the low-expression group and 14 of 23 (60.9%) in the high-expression group.

**Figure 4:**
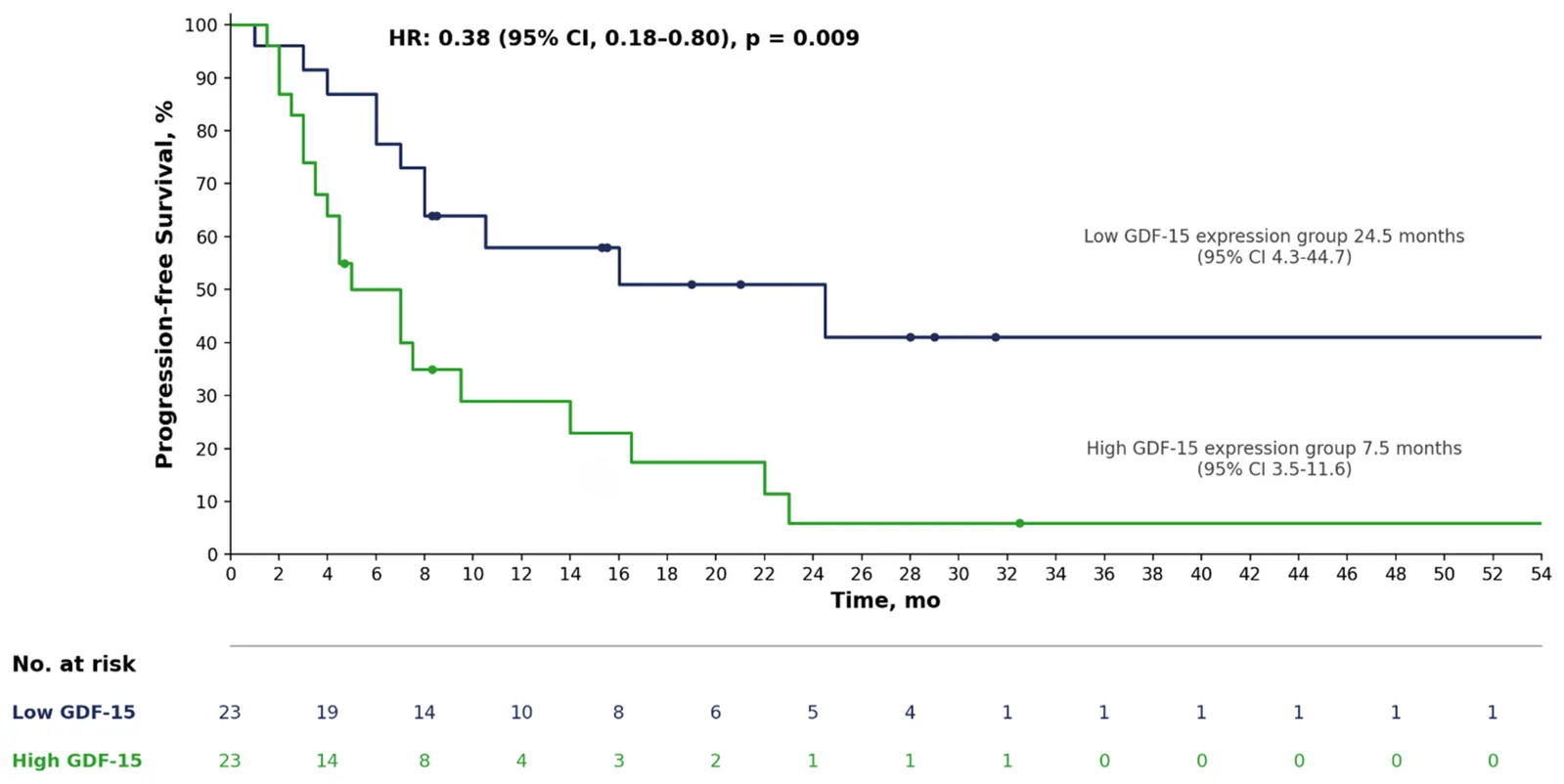
Progression-free survival by tumour GDF-15 expression. Kaplan–Meier curves; median 24.5 months with low expression versus 7.5 months with high expression (HR 0.38, 95% CI 0.18 to 0.80; *p* = 0.009).

After the start of second-line nivolumab, patients with low tumour GDF-15 expression had significantly longer PFS than those with high expression (24.5 months [95% CI, 4.3–44.7] vs. 7.5 months [95% CI, 3.5–11.6]; HR 0.38, 95% CI 0.18–0.80; *p* = 0.009; Figure 4). Age, sex, ECOG PS, and prior TKI were not significantly associated with PFS on univariable analysis (Table 2). GDF-15 expression was the only variable significantly associated with PFS (Table 2). Univariable Cox regression results for PFS are additionally presented graphically in Supplementary Figure S1.

### 3.4. Overall Survival

**Figure 5:**
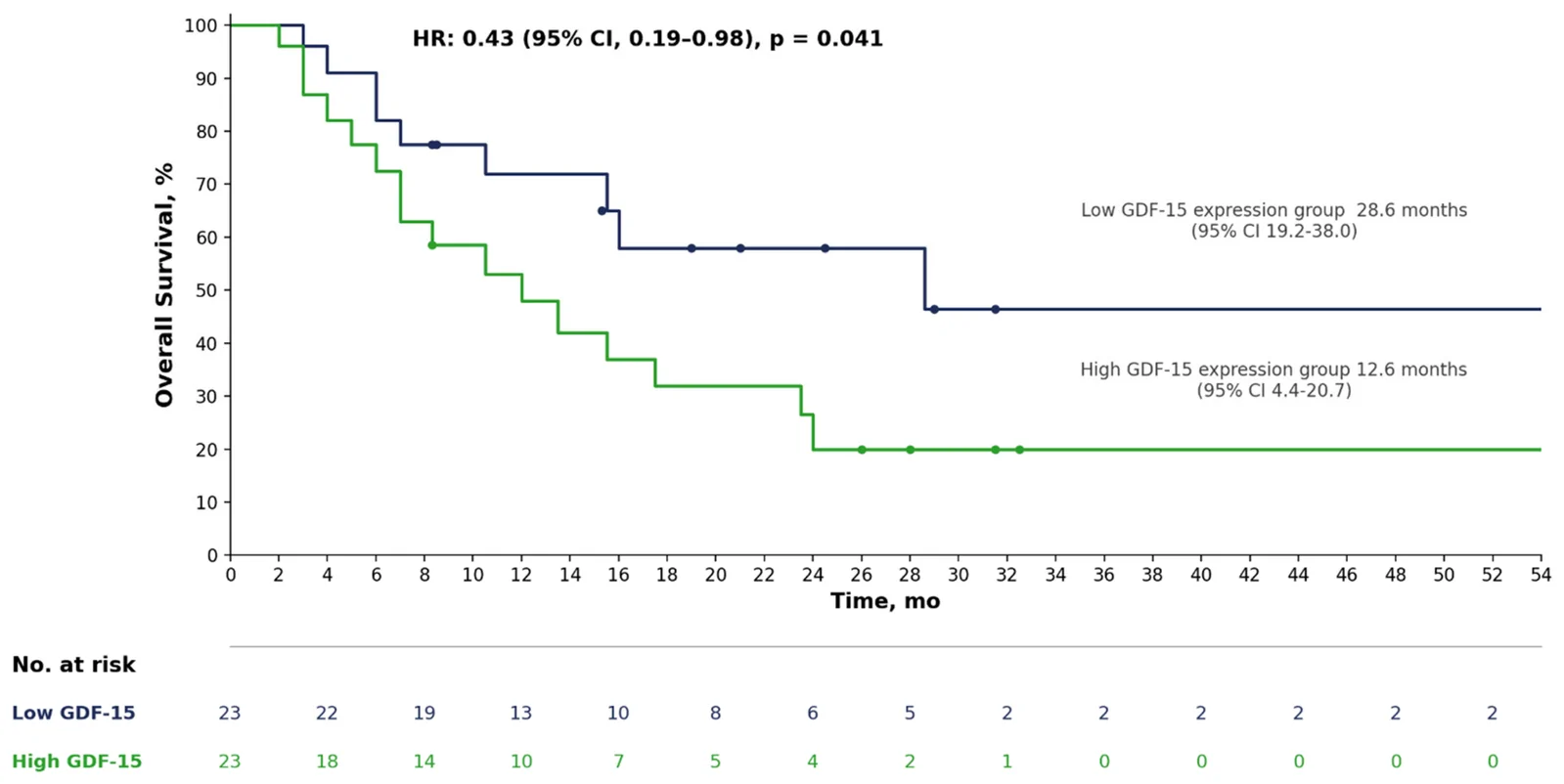
Overall survival by tumour GDF-15 expression. Kaplan–Meier curves; median 28.6 months with low expression versus 12.6 months with high expression (HR 0.43, 95% CI 0.19 to 0.98; *p* = 0.041).

During follow-up, 24 deaths occurred: 11 of 23 patients (47.8%) in the low-expression group and 13 of 23 (56.5%) in the high-expression group. Median OS for the whole cohort was 15.7 months (95% CI, 10.4 to 20.9). Patients with low GDF-15 expression had a median OS of 28.6 months (95% CI, 19.2 to 38.0) compared with 12.6 months (95% CI, 4.4 to 20.7) in the high-expression group (HR 0.43, 95% CI 0.19 to 0.98; *p* = 0.041; Figure 5). On univariable analysis, age ≥65 years was associated with shorter OS, whereas sex, ECOG PS, and prior TKI were not. After adjustment for age, low tumour GDF-15 expression remained associated with longer OS (HR 0.33, 95% CI 0.13–0.79; *p* = 0.011; Table 3). Univariable Cox regression results for OS are additionally presented graphically in Supplementary Figure S2.

### 3.5. Cancer-Associated Cachexia

Cancer-associated cachexia was identified in 18 patients (39%). Its frequency did not differ by tumour GDF-15 expression, occurring in 10 patients (43%) in the high-expression group and 8 patients (35%) in the low-expression group (*p* = 0.546).

## 4. Discussion

In this homogeneous cohort of patients with intermediate-risk clear-cell mRCC treated with second-line nivolumab, low tumour GDF-15 expression was consistently linked to better outcomes. Patients with low expression responded more often, remained progression-free for longer and survived longer, with the association with overall survival remaining significant after adjustment for age. The stronger association between low GDF-15 expression and OS after adjustment for age is consistent with a potential negative confounding effect of age, given the slightly older age distribution in the low-expression group. No statistically significant differences in baseline clinicopathological characteristics were observed between the two groups, although residual confounding inherent to retrospective studies cannot be excluded. Taken together, these findings support further investigation of tumour GDF-15 as a candidate prognostic biomarker in this clinical setting.

Reliable markers remain an unmet need in mRCC. Treatment decisions are largely guided by IMDC risk classification, a model built from patient and disease characteristics that says little about the tumour itself and offers limited help in anticipating response to immunotherapy. Tissue-based measures can capture some of the biological heterogeneity within the tumour microenvironment. Our findings suggest that tumour GDF-15 expression may provide additional prognostic information within a clinically homogeneous intermediate-risk population, although its incremental value beyond established clinical risk models was not formally tested. The practical appeal is that immunohistochemistry is inexpensive, uses archival material and is available in any pathology laboratory.

The biological rationale for these observations is reasonably well developed. GDF-15 shapes the tumour immune microenvironment through several mechanisms, including impaired dendritic cell maturation, weaker cytotoxic T-cell activation and a shift towards an immunosuppressive milieu [6,7]. Each of these would be expected to reduce the efficacy of PD-1 blockade. The GDFATHER study provided direct clinical support: neutralising GDF-15 with visugromab restored antitumour activity in patients whose disease had become refractory to anti-PD-1 or anti-PD-L1 therapy, and treatment was accompanied by increased T-cell infiltration and interferon-related signalling within tumours [11]. Our finding that patients with low tumour GDF-15 expression achieved both higher response rates and longer survival fits that model, and places the present cohort on the same biological axis as an actively developed therapeutic programme. These findings provide further rationale for prospective studies evaluating whether tissue GDF-15 expression may help identify patients most likely to benefit from therapeutic strategies targeting the GDF-15 pathway.

Earlier clinical work on GDF-15 in renal cell carcinoma has almost entirely used serum. Traeger et al. reported that raised serum concentrations accompanied advanced disease and shorter survival, identifying GDF-15 as a candidate marker in RCC [13]. Circulating concentrations are influenced by ageing, chronic kidney disease, chronic lung disease, heart failure and inflammation [5,15,16,17], which limits their specificity in an older population with substantial comorbidity. Tissue expression reports more directly on the tumour. To our knowledge, this is the first study to assess tumour GDF-15 expression specifically in patients with metastatic clear-cell RCC receiving nivolumab, and it extends the serum-based literature into the immunotherapy setting.

Tumour GDF-15 expression was not associated with cachexia in our cohort, even though high expression predicted worse outcomes. The same dissociation has been reported in non-small cell lung cancer [19]. A plausible explanation is that the two measures report on different compartments. Circulating GDF-15 reaches the brainstem GFRAL receptor and drives the anorexia and weight loss of cachexia, and it can originate from tissues other than the tumour, whereas intratumoural expression reflects local immune regulation. If that is correct, tissue GDF-15 is the more informative measure for outcome after immunotherapy, while serum GDF-15 remains the better test for identifying patients at risk of cachexia and candidates for cachexia-directed antibodies [12,20]. Previous studies have shown that cancer-associated weight loss and adverse body composition are associated with poorer outcomes following immune checkpoint inhibitor therapy [21,25].

The response data deserve emphasis. Most biomarker studies in RCC report survival endpoints alone, and radiological response receives less attention. Here, ORR, PFS and OS all favoured the low GDF-15 expression group, with a substantial absolute difference in ORR (57% versus 26%). The concordant direction of these findings strengthens the internal consistency of our observations. Nevertheless, these endpoints are biologically related and should not be interpreted as independent validation of the association. From a clinical perspective, a biomarker associated with radiological response may have greater practical utility than one associated with survival outcomes alone.

PD-L1 expression was not available because it is not assessed routinely in RCC. PD-L1 has shown limited prognostic and predictive value in renal cell carcinoma and is not currently used to select systemic therapy in this disease [26]. Nevertheless, prospective studies incorporating both PD-L1 and GDF-15 could determine whether their combined assessment provides complementary prognostic information beyond either marker alone. The absence of a direct PD-L1 comparison further supports the need for prospective studies evaluating GDF-15 alongside other tissue-based markers, particularly given its biological link to potentially targetable mechanisms of immune resistance. Recent reviews of the evolving biomarker landscape in RCC similarly emphasise the continuing lack of clinically validated predictive biomarkers and the need to integrate emerging tissue and molecular markers into prospective studies [27,28].

Several limitations should be acknowledged. The retrospective design, modest sample size, and limited number of survival events reduce the precision of our estimates, as reflected by the relatively wide confidence intervals, and call for cautious interpretation. This also increases the risk of overfitting in the multivariable Cox models; therefore, the adjusted estimates should be interpreted cautiously. The single-centre design and absence of an independent validation cohort further limit the robustness and generalisability of the findings. Selection bias also cannot be excluded, as eligibility required available archival tumour tissue, and patients without suitable tissue could not be evaluated. Restricting the cohort to intermediate-risk patients receiving second-line nivolumab monotherapy produced a clinically homogeneous population, but substantially limits generalisability to favourable- and poor-risk disease, contemporary first-line ICI-based combination regimens, and other immune checkpoint inhibitors. National reimbursement rules confine nivolumab to single-agent second-line use in mRCC, which is why all patients were treated in this way. Although the IHC cut-off was pre-specified based on previous GDF-15 tissue studies and was not derived from outcomes in the present cohort, an RCC-specific threshold has not yet been established or externally validated. GDF-15 staining was independently evaluated by two pathologists with excellent interobserver agreement; however, no external central pathology review was performed. Cachexia was evaluated using serial body weight and BMI measurements without standardised radiological assessment of skeletal muscle mass. Consequently, sarcopenia-based diagnostic criteria could not be incorporated. In addition, the retrospective assessment schedule did not permit reliable determination of the exact onset of cachexia, precluding valid time-dependent survival analyses. Finally, because every patient received nivolumab, our findings show prognostic rather than predictive value. Establishing whether GDF-15 identifies patients who derive greater benefit from checkpoint inhibition than from other systemic therapies will require prospective comparative studies.

## 5. Conclusions

Low tumour GDF-15 expression was associated with better objective response and longer progression-free and overall survival in patients with intermediate-risk metastatic clear-cell renal cell carcinoma treated with second-line nivolumab, with the association with overall survival remaining significant after adjustment for age. Given the small, selected cohort and the specific second-line treatment setting, these findings should be considered hypothesis-generating and require validation in larger, independent cohorts, including patients receiving contemporary first-line ICI-based combination regimens. Tissue GDF-15 may therefore represent a promising, inexpensive candidate for further evaluation as a prognostic biomarker and adds a further rationale for prospective studies of the GDF-15 pathway as both a marker of outcome and a therapeutic target in renal cell carcinoma. Studies that combine tissue GDF-15 with established clinical models and emerging molecular markers may help refine individualised treatment for this disease.

## Figures and Tables

**Table 1:** Demographic and clinicopathological characteristics according to tumour GDF-15 expression.

Characteristic	Total (*n* = 46)	Low GDF-15 (*n* = 23)	High GDF-15 (*n* = 23)	*p* Value
**Age, median (range), years**	62.7 (42–78)	63.4 (42–74)	60.2 (44–78)	0.431
**Sex,** ***n*** **(%)**				0.713
Male	37 (80)	18 (78)	19 (83)	
Female	9 (20)	5 (22)	4 (17)	
**ECOG PS,** ***n*** **(%)**				0.459
0	9 (20)	3 (13)	6 (26)	
1	37 (80)	20 (87)	17 (74)	
**Chronic kidney disease,** ***n*** **(%)**				0.550
Yes	3 (7)	2 (9)	1 (4)	
No	43 (93)	21 (91)	22 (96)	
**Prior TKI,** ***n*** **(%)**				0.216
Sunitinib	30 (65)	17 (74)	13 (57)	
Pazopanib	16 (35)	6 (26)	10 (43)	
**Cachexia,** ***n*** **(%)**				0.546
Yes	18 (39)	8 (35)	10 (43)	
No	28 (61)	15 (65)	13 (57)	
**Disease distribution,** ***n*** **(%)**				0.659
Bone only	6 (13)	2 (9)	4 (18)	
Visceral only	27 (59)	15 (65)	12 (52)	
Multiple sites	13 (28)	6 (26)	7 (30)	
**Best overall response,** ***n*** **(%)**				0.033
Complete response	4 (8)	3 (13)	1 (4)	
Partial response	15 (33)	10 (44)	5 (22)	
Stable disease	9 (20)	4 (17)	5 (22)	
Progressive disease	18 (39)	6 (26)	12 (52)	
**Subsequent systemic therapy,** ***n*** **(%)**				0.765
Yes	19 (41)	9 (39)	10 (43)	
No	27 (59)	14 (61)	13 (57)	

Data are n (%) unless stated otherwise. Abbreviations: GDF-15, growth differentiation factor-15; ECOG PS, Eastern Cooperative Oncology Group performance status; TKI, tyrosine kinase inhibitor.

**Table 2:** Univariable analysis for progression-free survival.

Characteristic	*n* (%)	mPFS, Months	Univariable HR (95% CI); *p* Value
Age			
<65 years	27 (59)	16.2	1.00 (reference)
≥65 years	19 (41)	6.8	1.84 (0.88–3.86); 0.111
Sex			
Female	9 (20)	6.8	1.00 (reference)
Male	37 (80)	9.3	0.88 (0.40–1.93); 0.750
ECOG PS			
0	9 (20)	9.3	1.00 (reference)
1	37 (80)	7.5	1.21 (0.64–2.27); 0.547
Prior TKI			
Sunitinib	30 (65)	7.5	1.00 (reference)
Pazopanib	16 (35)	10.6	0.87 (0.40–1.87); 0.720
GDF-15			
High	23 (50)	7.5	1.00 (reference)
Low	**23 (50)**	**24.5**	**0.38 (0.18–0.80); 0.009**

mPFS, median progression-free survival; HR, hazard ratio; CI, confidence interval; ECOG PS, Eastern Cooperative Oncology Group performance status; TKI, tyrosine kinase inhibitor. No multivariable Cox model was constructed because GDF-15 was the only variable meeting the predefined inclusion criterion (*p* < 0.10) on univariable analysis. Bold values indicate statistical significance (*p* < 0.05).

**Table 3:** Univariable and multivariable analysis for overall survival.

Characteristic	n (%)	mOS, Months	Univariable HR (95% CI); *p*	Multivariable HR (95% CI); *p* Value
Age				
<65 years	27 (59)	24.5	1.00 (reference)	1.00 (reference)
≥65 years	19 (41)	10.4	**2.37 (1.04–5.39); 0.034**	**3.56 (1.40–9.01); 0.007**
Sex				
Male	37 (80)	17.5	1.00 (reference)	-
Female	9 (20)	15.7	1.08 (0.41–2.90); 0.872	-
ECOG PS				
0	9 (20)	23.5	1.00 (reference)	-
1	37 (80)	15.2	1.44 (0.49–4.24); 0.498	-
Prior TKI				
Sunitinib	30 (65)	15.2	1.00 (reference)	-
Pazopanib	16 (35)	17.5	0.99 (0.44–2.26); 0.996	-
GDF-15				
High	23 (50)	12.6	1.00 (reference)	1.00 (reference)
Low	23 (50)	28.6	**0.43 (0.19–0.98); 0.041**	**0.33 (0.13–0.79); 0.011**

mOS, median overall survival; HR, hazard ratio; CI, confidence interval; ECOG PS, Eastern Cooperative Oncology Group performance status; TKI, tyrosine kinase inhibitor. The multivariable model included variables with *p* < 0.10 on univariable analysis. Given the limited number of deaths (*n* = 24), the multivariable model should be considered exploratory. Bold values indicate statistical significance (*p* < 0.05).

## Data Availability

The data supporting the findings of this study are available from the corresponding author on reasonable request. The data are not publicly available because they contain information that could compromise patient privacy.

## Supplementary Materials

The following supporting information can be downloaded at: https://www.mdpi.com/article/10.3390/curroncol33090531/s1, Figure S1: Univariable Cox regression for progression-free survival; Figure S2: Univariable Cox regression for overall survival.

## Abbreviations

CI, confidence interval; CT, computed tomography; ECOG PS, Eastern Cooperative Oncology Group performance status; GDF-15, growth differentiation factor-15; GFRAL, glial cell-derived neurotrophic factor family receptor alpha-like; HR, hazard ratio; ICI, immune checkpoint inhibitor; IMDC, International Metastatic Renal Cell Carcinoma Database Consortium; mRCC, metastatic renal cell carcinoma; ORR, objective response rate; OS, overall survival; PD-L1, programmed death-ligand 1; PET, positron emission tomography; PFS, progression-free survival; RECIST, Response Evaluation Criteria in Solid Tumours; TKI, tyrosine kinase inhibitor.

## References

[1] Escudier B., Porta C., Schmidinger M., Rioux-Leclercq N., Bex A., Khoo V., Grünwald V., Gillessen S., Horwich A. (2019). Renal cell carcinoma: ESMO Clinical Practice Guidelines for diagnosis, treatment and follow-up. Ann. Oncol..

[2] Heng D.Y., Xie W., Regan M.M., Warren M.A., Golshayan A.R., Sahi C., Eigl B.J., Ruether J.D., Cheng T., North S. (2009). Prognostic factors for overall survival in patients with metastatic renal cell carcinoma treated with vascular endothelial growth factor-targeted agents: Results from a large, multicenter study. J. Clin. Oncol..

[3] Motzer R.J., Bacik J., Murphy B.A., Russo P., Mazumdar M. (2002). Interferon-alfa as a comparative treatment for clinical trials of new therapies against advanced renal cell carcinoma. J. Clin. Oncol..

[4] Holland L., Bhanvadia R., Ibeziako N., Taylor J., Gerlt D., Chaplin I., Bagrodia A., Desai N., Gaston K., Lotan Y. (2024). Socioeconomic and demographic disparities in immunotherapy utilization for advanced kidney and bladder cancer. Urol. Oncol..

[5] Breit S.N., Johnen H., Cook A.D., Tsai V.W.W., Mohammad M.G., Kuffner T., Zhang H.P., Marquis C.P., Jiang L., Lockwood G. (2011). The TGF-beta superfamily cytokine, MIC-1/GDF15: A pleotrophic cytokine with roles in inflammation, cancer and metabolism. Growth Factors.

[6] Wischhusen J., Melero I., Fridman W.H. (2020). Growth/differentiation factor-15 (GDF-15): From biomarker to novel targetable immune checkpoint. Front. Immunol..

[7] Zhou Z., Li W., Song Y., Wang L., Zhang K., Yang J., Zhang W., Su H., Zhang Y. (2013). Growth differentiation factor-15 suppresses maturation and function of dendritic cells and inhibits tumor-specific immune response. PLoS ONE.

[8] Emmerson P.J., Wang F., Du Y., Liu Q., Pickard R.T., Gonciarz M.D., Coskun T., Hamang M.J., Sindelar D.K., Ballman K.K. (2017). The metabolic effects of GDF15 are mediated by the orphan receptor GFRAL. Nat. Med..

[9] Hsu J.Y., Crawley S., Chen M., Ayupova D.A., Lindhout D.A., Higbee J., Kutach A., Joo W., Gao Z., Fu D. (2017). Non-homeostatic body weight regulation through a brainstem-restricted receptor for GDF15. Nature.

[10] Breit S.N., Brown D.A., Tsai V.W. (2021). The GDF15-GFRAL pathway in health and metabolic disease: Friend or foe?. Annu. Rev. Physiol..

[11] Melero I., de Miguel Luken M., de Velasco G., Garralda E., Martín-Liberal J., Joerger M., Alonso G., Goebeler M.E., Schuler M., König D. (2025). Neutralizing GDF-15 can overcome anti-PD-1 and anti-PD-L1 resistance in solid tumours. Nature.

[12] Groarke J.D., Crawford J., Collins S.M., Lubaczewski S., Roeland E.J., Naito T., Hendifar A.E., Fallon M., Takayama K., Asmis T. (2024). Ponsegromab for the treatment of cancer cachexia. N. Engl. J. Med..

[13] Traeger L., Ellermann I., Wiethoff H., Ihbe J., Gallitz I., Eveslage M., Moritz R., Herrmann E., Schrader A.J., Steinbicker A.U. (2019). Serum hepcidin and GDF-15 levels as prognostic markers in urothelial carcinoma of the upper urinary tract and renal cell carcinoma. BMC Cancer.

[14] Welsh J.B., Sapinoso L.M., Kern S.G., Brown D.A., Liu T., Bauskin A.R., Ward R.L., Hawkins N.J., Quinn D.I., Russell P.J. (2003). Large-scale delineation of secreted protein biomarkers overexpressed in cancer tissue and serum. Proc. Natl. Acad. Sci. USA.

[15] Nair V., Robinson-Cohen C., Smith M.R., Bellovich K.A., Bhat Z.Y., Bobadilla M., Brosius F., de Boer I.H., Essioux L., Formentini I. (2017). Growth differentiation factor-15 and risk of CKD progression. J. Am. Soc. Nephrol..

[16] Wan Y., Fu J. (2024). GDF15 as a key disease target and biomarker: Linking chronic lung diseases and ageing. Mol. Cell. Biochem..

[17] Bouabdallaoui N., Claggett B., Zile M.R., McMurray J.J.V., O’Meara E., Packer M., Prescott M.F., Swedberg K., Solomon S.D., Rouleau J.L. (2018). Growth differentiation factor-15 is not modified by sacubitril/valsartan and is an independent marker of risk in patients with heart failure and reduced ejection fraction: The PARADIGM-HF trial. Eur. J. Heart Fail..

[18] Akdogan O., Ogut B., Sutcuoglu O., Sert A., Gurler F., Akyurek N., Ozdemir N., Ozet A., Yazici O. (2024). The impact of the expression level of growth differentiation factor 15 in tumor tissue on the response to immunotherapy in non-small cell lung cancer. BMC Cancer.

[19] Morita-Tanaka S., Miyagawa-Hayashino A., Yamada T., Matsui Y., Morimoto K., Hiranuma O., Masuzawa N., Yoshimura A., Iwasaku M., Tokuda S. (2023). Significance of localized expression of full-length growth differentiation factor-15 in cachexia of advanced non-small cell lung cancer. Support. Care Cancer.

[20] Lerner L., Hayes T.G., Tao N., Krieger B., Feng B., Wu Z., Nicoletti R., Chiu M.I., Gyuris J., Garcia J.M. (2015). Plasma growth differentiation factor 15 is associated with weight loss and mortality in cancer patients. J. Cachexia Sarcopenia Muscle.

[21] Madeddu C., Busquets S., Donisi C., Lai E., Pretta A., López-Soriano F.J., Argilés J.M., Scartozzi M., Macciò A. (2023). Effect of cancer-related cachexia and associated changes in nutritional status, inflammatory status, and muscle mass on immunotherapy efficacy and survival in patients with advanced non-small cell lung cancer. Cancers.

[22] Fearon K., Strasser F., Anker S.D., Bosaeus I., Bruera E., Fainsinger R.L., Jatoi A., Loprinzi C., MacDonald N., Mantovani G. (2011). Definition and classification of cancer cachexia: An international consensus. Lancet Oncol..

[23] Evans W.J., Morley J.E., Argilés J., Bales C., Baracos V., Guttridge D., Jatoi A., Kalantar-Zadeh K., Lochs H., Mantovani G. (2008). Cachexia: A new definition. Clin. Nutr..

[24] Eisenhauer E.A., Therasse P., Bogaerts J., Schwartz L.H., Sargent D., Ford R., Dancey J., Arbuck S., Gwyther S., Mooney M. (2009). New response evaluation criteria in solid tumours: Revised RECIST guideline (version 1.1). Eur. J. Cancer.

[25] Al-Sawaf O., Weiss J., Skrzypski M., Lam J.M., Karasaki T., Zambrana F., Kidd A.C., Frankell A.M., Watkins T.B.K., Martínez-Ruiz C. (2023). Body composition and lung cancer-associated cachexia in TRACERx. Nat. Med..

[26] Powles T., Albiges L., Bex A., Compérat E., Grünwald V., Kanesvaran R., Kitamura H., McKay R., Porta C., Procopio G. (2024). Renal cell carcinoma: ESMO Clinical Practice Guideline for diagnosis, treatment and follow-up. Ann. Oncol..

[27] McKinnon M.B., Rini B.I., Haake S.M. (2025). Biomarker-informed care for patients with renal cell carcinoma. Nat. Cancer.

[28] Bakouny Z., Hakimi A.A., Reznik E., Motzer R.J. (2026). Biomarkers for renal cell carcinoma—A pragmatic approach. Nat. Rev. Urol..

